# Effect of pre-operative hypoxemia on the occurrence and outcomes of post-operative ARDS in Stanford type a aortic dissection patients

**DOI:** 10.1186/s12931-023-02457-8

**Published:** 2023-06-17

**Authors:** Teng Cai, Zhonghua Fei, Hongsheng Liu, Xinmei Liu, Zhansheng Hu

**Affiliations:** 1 Suzhou Medical College of Soochow University, Suzhou, 215123 China; 2 Cardiac Intensive Care Unit, Affiliated Hospital of Jining Medical University, Jining, 272000 China; 3 Department of Intensive Care Unit, The First Affiliated Hospital of Jinzhou Medical University, Jinzhou, 121000 China

**Keywords:** Pre-operative hypoxemia, Acute respiratory distress syndrome (ARDS), Stanford type A aortic dissection (AAD)

## Abstract

**Background:**

Pre-operative and post-operative hypoxemia are frequent complications of Stanford type A aortic dissection (AAD). This study explored the effect of pre-operative hypoxemia on the occurrence and outcome of post-operative acute respiratory distress syndrome (ARDS) in AAD.

**Method:**

A total of 238 patients who underwent surgical treatment for AAD between 2016 and 2021 were enrolled. Logistic regression analysis was conducted to investigate the effect of pre-operative hypoxemia on post-operative simple hypoxemia and ARDS. Post-operative ARDS patients were divided into pre-operative normal oxygenation group and pre-operative hypoxemia group that were compared for clinical outcomes. Post-operative ARDS patients with pre-operative normal oxygenation were classified as the real ARDS group. Post-operative ARDS patients with pre-operative hypoxemia, post-operative simple hypoxemia, and post-operative normal oxygenation were classified as the non-ARDS group. Outcomes of real ARDS and non-ARDS groups were compared.

**Result:**

Logistic regression analysis showed that pre-operative hypoxemia was positively associated with the risk of post-operative simple hypoxemia (odds ratios (OR) = 4.81, 95% confidence interval (CI): 1.67–13.81) and post-operative ARDS (OR = 8.514, 95% CI: 2.64–27.47) after adjusting for the confounders. The post-operative ARDS with pre-operative normal oxygenation group had significantly higher lactate, APACHEII score and longer mechanical ventilation time than the post-operative ARDS with pre-operative hypoxemia group (P < 0.05). Pre-operative the risk of death within 30 days after discharge was slightly higher in ARDS patients with pre-operative normal oxygenation than in ARDS patients with pre-operative hypoxemia, but there was no statistical difference(log-rank test, P = 0.051). The incidence of AKI and cerebral infarction, lactate, APACHEII score, mechanical ventilation time, intensive care unit and post-operative hospital stay, and mortality with 30 days after discharge were significantly higher in the real ARDS group than in the non-ARDS group (P < 0.05). After adjusting for confounding factors in the Cox survival analysis, the risk of death within 30 days after discharge was significantly higher in the real ARDS group than in the non-ARDS group (hazard ratio(HR): 4.633, 95% CI: 1.012–21.202, P < 0.05).

**Conclusion:**

Preoperative hypoxemia is an independent risk factor for post-operative simple hypoxemia and ARDS. Post-operative ARDS with pre-operative normal oxygenation was the real ARDS, which was more severe and associated with a higher risk of death after surgery.

**Supplementary Information:**

The online version contains supplementary material available at 10.1186/s12931-023-02457-8.

## Background

Stanford type A aortic dissection (AAD) is caused by the tearing of the intima of the ascending aorta that leads to the entry of the blood into the aortic middle layer, resulting in the formation of hematoma in the aorta [1]. AAD has a morbidity of about 2.6 to 3.6 per 100,000 cases [2]. Once the diagnosis is confirmed, surgical treatment is imperative, but can be difficult and traumatic and has many post-operative complications [3, 4]. At present, AAD-associated mortality is up to 18% [4, 5]. Patients with AAD frequently experience pre-operative and post-operative hypoxemia, particularly, post-operative hypoxemia that has a very high incidence and leads to prolonged mechanical ventilation, increased risk of ventilator-related lung injury and nosocomial infection, and even life-threatening events [6–9]. The current understanding of the mechanism of pre-operative and post-operative hypoxemia in patients with aortic dissection is unclear.

Acute respiratory distress syndrome (ARDS) is a clinically heterogeneous syndrome characterized by refractory hypoxemia and respiratory distress [10–13]. The European Society for Critical Care medicine and the American Thoracic Society jointly published the Berlin Definition of ARDS in 2012 [14] that described ARDS diagnosis based on four aspects, that were respiratory symptoms, oxygenation index (OI, partial pressure of arterial oxygen/fraction of inspired oxygen(PaO_2_/FiO_2_)), origin of pulmonary edema, and chest imaging. ARDS has the characteristics of acute onset and is associated with a fatality rate as high as 21–50% [15–17]. The incidence of post-operative hypoxemia is high in AAD [6, 18] and may complicate the diagnosis of ARDS after surgery, leading to a high false positive result of ARDS. Multiple studies have shown that patients with AAD have a high incidence of post-operative ARDS, but there is no increased risk of mortality or early death [19, 20].

We speculate that hypoxemia, as a confounding factor, may affect previous study results on the clinical outcome of ARDS. In this study, we explore the relationship between pre-operative hypoxemia and the risk of post-operative simple hypoxemia and ARDS, as well as the effect of pre-operative hypoxemia on the severity of ARDS and the risk of death. The aim is to find the real ARDS after AAD surgery to improve our understanding of ARDS after AAD surgery and better interpret the results of previous studies.

## Methods

### Study design

**Fig. 1:**
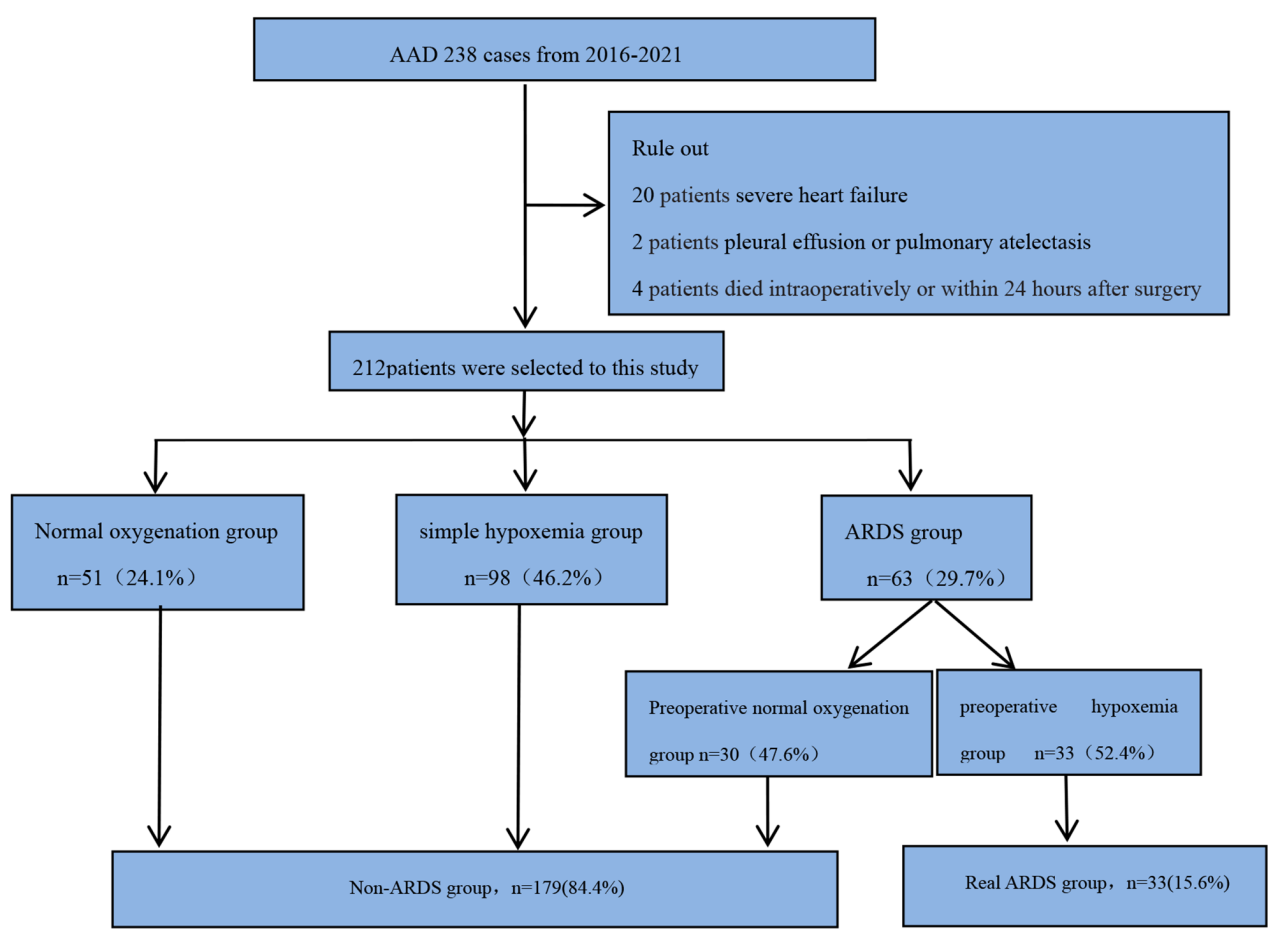
Flow chart of the study

This was a retrospective single-center case-control study. To explore the relationship between pre-operative hypoxemia and the risk of post-operative ARDS, all subjects were divided into three groups based on post-operative oxygenation and Berlin Definition [14] as follows: (1) normal oxygenation group (post-operative OI > 300), (2) simple hypoxemia group (post-operative OI < 300, but other conditions of the Berlin Definition are not met), (3) ARDS group (based on the Berlin Definition). According to pre-operative OI, the post-operative ARDS group was divided into ARDS with pre-operative normal oxygenation and ARDS with pre-operative hypoxemia. The clinical outcomes of the two groups were analyzed (Fig. 1).

Post-operative ARDS patients with pre-operative normal oxygenation were classified as the real ARDS group. Post-operative ARDS patients with pre-operative hypoxemia, post-operative simple hypoxemia patients and post-operative normal oxygenation patients were classified as the non-ARDS group. Outcomes were compared between the real ARDS group and non-ARDS group (Fig. 1). The study protocol was approved by the ethics board of the Affiliated Hospital of Jining Medical University(NO.2022C059). Because of the retrospective nature of the study, individual consent was not required.

### Patients

A total of 238 patients were diagnosed aortic dissection and underwent surgical treatment in the Cardiac Intensive Care Unit at the Affiliated Hospital of Jining Medical University between January 2016 and December 2021. 26 patients excluded: (1) 20 patients with severe heart failure (NYHA classification level ≥ III grade) that were recognized before the diagnosis of ARDS; (2) 2 patients with massive pleural effusion or pulmonary atelectasis before the diagnosis of ARDS; (3) 4 patients who died intraoperatively or within 24 h after surgery because their post-operative clinical data was severely missing. 212 patients were included in the analysis.

### Definition of events

1. ARDS: Based on the Berlin Definition for ARDS [14](supplementary Table 1), it is necessary to exclude cardiogenic pulmonary edema and evaluate scope of lung injury. Unfortunately, PAWP was not monitored in the patients. However, bedside echocardiography was performed for patients with suspected ARDS to rule out cardiogenic pulmonary edema as much as possible. Bedside chest radiographs were performed on the first day after surgery for suspected ARDS to evaluate the scope of lung injury.
2. Hypoxemia: OI < 300 over 24 h was defined as hypoxemia, excluding patients with transient hypoxemia.
3. Perioperative blood loss and perioperative transfusion: Intraoperative and post-operative blood loss and blood transfusion volume within 24 h.
4. APACHE II score: The APACHE II score within the first 24 h from surgery.
5. Acute kidney injury(AKI): According to the KDIGO criteria, perioperative AKI is defined by the basis of postoperative serum creatinine with more than 50% increase or 0.3 mg/dl increase in serum creatinine.The pre-operative serum creatinine was defined as the baseline serum creatinine [21].
6. Hepatic dysfunction: Alanine aminotransferase(ALT)>200U/L and bilirubin>50mmol/L with or without manifestations of hepatic dysfunction [22].
7. Lactate: Maximum value with the 48 h after surgery.
8. Perioperative fluid load: Perioperative fluid load with the 48 h after surgery.

### Data collection

All data were collected from the electronic medical record system of the Affiliated Hospital of Jining Medical University. Two patients were transferred to another hospital for treatment and three patients were discharged for hospice care. To clarify the situation of these patients, we followed up the survival of all the subjects within 30 days after discharge by telephone.

### Missing data

If pre-operative PaO_2_ was missing, pre-operative peripheral capillary oxygen saturation (SPO_2_)/FiO_2_ was used to determine patient oxygenation [23]. Pre-operative ALT data were missing for 17 patients (8.0%). All of these patients had ALT results on post-operative day 1 and one patient had ALT elevation. Pre-operative bilirubin data were missing for 18 patients (8.5%). All of these patients had bilirubin results on post-operative day 1; one patient had elevated bilirubin. Pre-operative creatinine data were missing for five patients (2.4%). All of these patients had creatinine results on post-operative day 1 and one patient had creatinine elevation. ALT, bilirubin, and creatinine deficiency values were replaced by medians, because these data didn’t conform to a normal distribution. All other data were complete.

### Statistical analysis

In general, we need to ensure that the ratio of potential independent variables to outcome events is 1/10 [24]. In this study, there were seven potential independent variables, and the ratio of potential independent variables to outcome events was 1.11/10.

All data were statistically analyzed by SPSS26. All statistical tests were two-sided, and P < 0.05 was considered statistically significant. Quantitative variables were described as the mean± (standard deviation [SD]) or the median (interquartile range [IQR]) as appropriate. The qualitative variables were described as frequency (percentage). Among the three groups, continuous variables were compared using the univariate analysis of variance (ANOVA) or non-parametric tests. LSD-t was used for post-hoc comparison. Between the two groups, continuous variables were compared using the *t*-test for normal distribution or Mann-Whitney’s U test for skewed distribution. Chi-square (x^2^) test or Fisher’s exact test was used for analysis of qualitative variables. Chi-square segmentation was used for multiple comparisons.

Variables with P<0.1 in the the univariate analysis were included in the multivariable model. The relationships among pre-operative hypoxemia and the risk of post-operative simple hypoxemia and ARDS were assessed by the multivariate logistic regression analysis by adopting a forward stepwise process. Univariable logistic regression was used to show the unadjusted associations. Perioperative blood loss and perioperative transfusion were highly correlated; hence, only perioperative blood loss was included in the multivariate logistic regression analysis to avoid any problem of collinearity.

The Kaplan-Meier survival analysis was used to draw survival curves. The Cox proportional hazard analysis was performed to adjust for confounders.

## Results

A total of 238 patients with AAD were enrolled; 26 patients were excluded and 212 patients were included in this study (Fig. 1). Seventy-six of 212 (35.8%) patients had pre-operative hypoxemia, 98 (46.2%) developed post-operative simple hypoxemia, and 63 (29.7%) developed post-operative ARDS. The median age of all the patients was 53 years (44.3–62.0), and 149 (70.3%) were male. The majority of the patients (163 [76.9%]) had hypertension, and the time from symptom onset to operation was 19 h (11.0-53.8)(Table 1).

### Pre-operative hypoxemia was associated with the risk of post-operative simple hypoxemia and ARDS

Univariate ANOVA or non-parametric tests (P < 0.1) showed that body mass index (BMI), hypertension, onset to operation time, pre-operative leukocyte, operation time, surgical method.

and perioperative blood loss were the potential factors associated with the risk of post-operative simple hypoxemia and ARDS (Table 1). We constructed three models to analyze the independent effects of pre-operative hypoxemia on the risk of post-operative simple hypoxemia and post-operative ARDS. In the unadjusted model I, pre-operative hypoxemia showed a positive correlation with the risk of post-operative simple hypoxemia (OR 5.17, 95% CI: 2.02–13.27) and post-operative ARDS (OR 6.2, 95% CI: 2.55–18.27). In model II (adjusted for BMI and hypertension), the result remained significant (postoperative simple hypoxemia, OR: 4.00; 95% CI: 1.53–10.58, post-operative ARDS, OR: 4.70; 95% CI: 1.67–13.25). In model III (adjusted for BMI, hypertension, onset to operation time, pre-operative leukocyte, operation time, surgical method and perioperative blood loss), the result still remained significant (post-operative simple hypoxemia, OR: 4.81; CI: 1.67–13.81;post-operative ARDS, OR: 8.51;CI:2.64–27.47)(Table 2).

### Clinical outcomes of normal oxygenation group, simple hypoxia group, and ARDS group

**Fig. 2:**
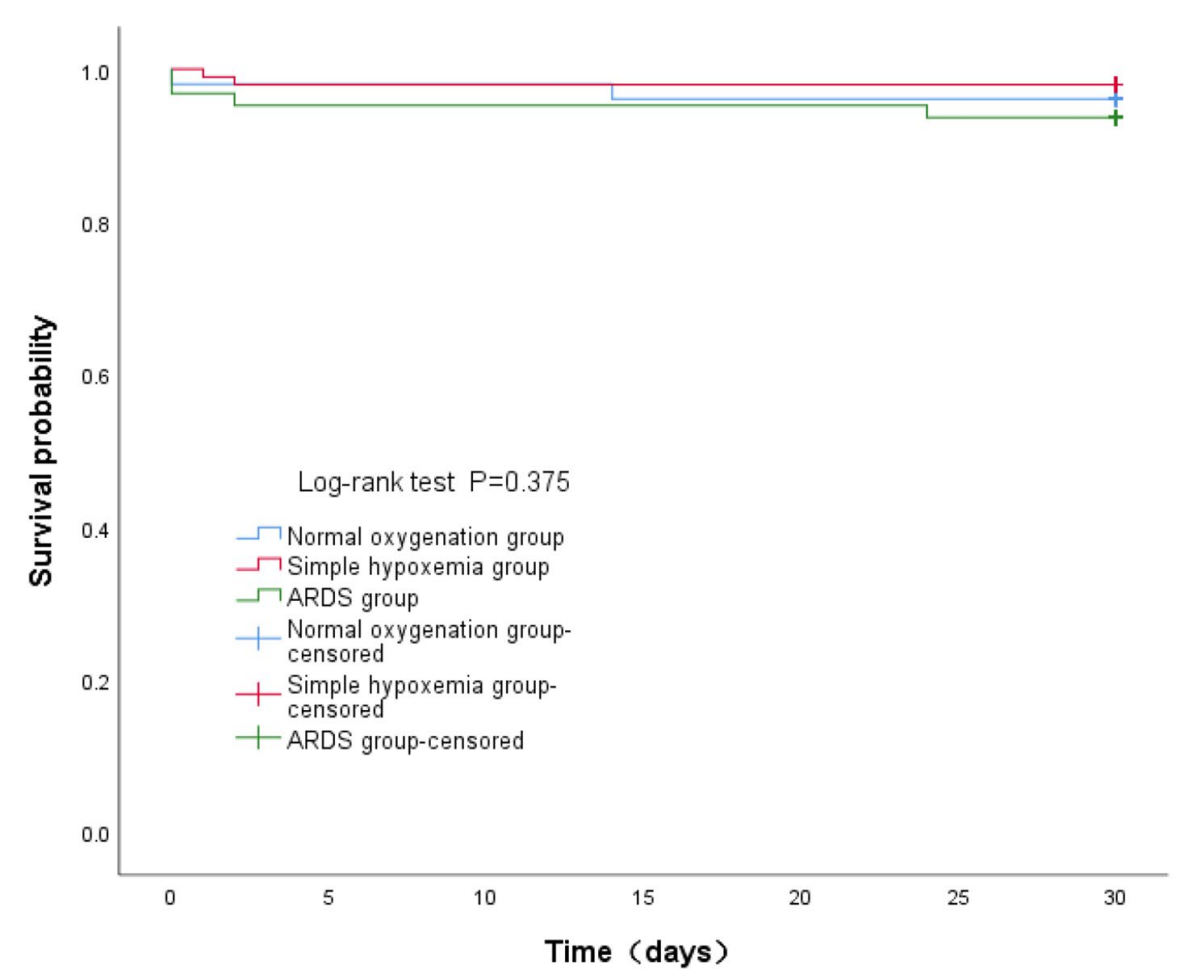
Comparison of the risk of death within 30 days after discharge in the normal oxygenation group, simple hypoxia group, and ARDS group

The APACHE II score, lactate, incidence of AKI were significantly higher and mechanical ventilation time, ICU stay, and post-operative hospital stay were significantly longer in the ARDS group (17.0 score [14.0–21.0], 4.6mmol/L(3.1-7.0), 23(36.5%), 86.0 h [57.3–158.0], 7 days [6-11.5], 18.0 days [14.0-24.5], respectively) than in the normal oxygenation group (12.0 score [11.0–14.0], 2.8mmol/L(2.0-3.6), 4(7.8%), 36.5 h [23.8–45.0], 4.0 days [3.0–6.0], 15.0 days [11.0–18.0], respectively) and simple hypoxia group (14.0 score [12.0–16.0], 3.3mmol/L(2.3–4.9), 19(19.4%), 40.0 h [30.8–57.1], 4 days [3.0-5.3], 13.0 days [11.0-17.3], respectively) (P < 0.05), but the values were not significantly different between the normal oxygenation group and simple hypoxia group (P > 0.05)(Table 3). Other results are shown in Table 3. There was no significant difference in the risk of death within 30 days after discharge among the three groups (log-rank test, P > 0.05; Fig. 2).

### Comparison between ARDS patients with pre-operative normal oxygenation and pre-operative hypoxemia

**Fig. 3:**
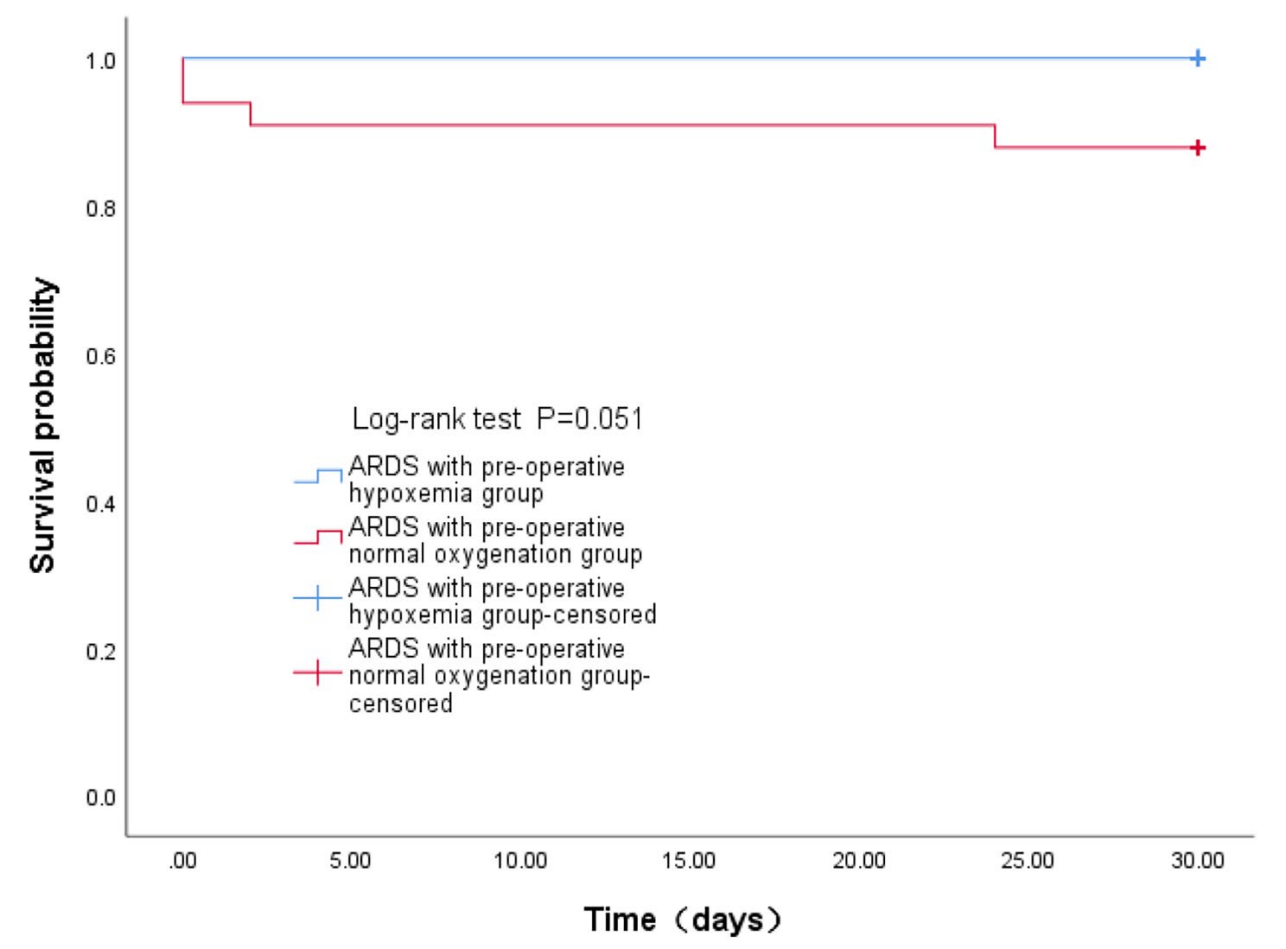
Comparison of the risk of death within 30 days after discharge between ARDS with pre-operative normal oxygenation group and ARDS with pre-operative hypoxemia group

There were no significant differences in the clinical characteristics and perioperative data, except for the perioperative blood loss, between ARDS patients with pre-operative normal oxygenation and pre-operative hypoxemia (P > 0.05). Perioperative blood loss, lactate and APACHE II score were significantly higher, and the mechanical ventilation time was significantly longer in ARDS patients with pre-operative normal oxygenation (3621.0mL[2393.0-7575.0], 4.8mmol/L(3.4–8.4), 18.0 score [14.5–24.0], 117.0 h [82.0-178.0], respectively) than in ARDS patients with pre-operative hypoxemia (2492.0 mL [2010.8–3732.0], 3.8mmol/L(2.8–5.6), 16.0 score [14.0–18.0], 70.5 h [42.0-122.3], respectively). The risk of death within 30 days after discharge was slightly higher in ARDS patients with pre-operative normal oxygenation than ARDS patients with pre-operative hypoxemia, but there was no statistical difference(log-rank test, P = 0.051,Fig. 3) Other complete results are shown in Tables 4 and 5.

### Comparison between the real ARDS and non-ARDS groups

**Fig. 4:**
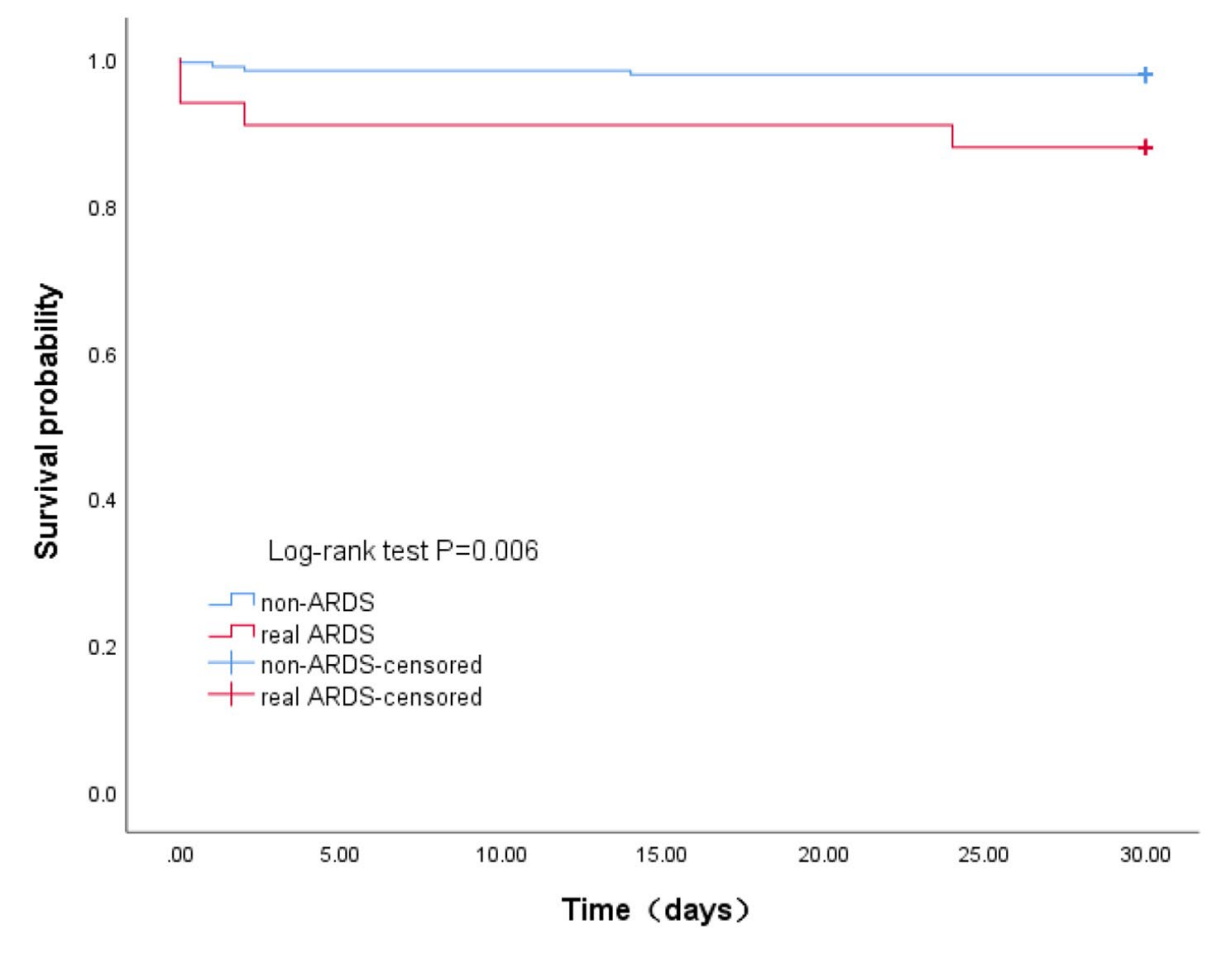
Comparison of the risk of death within 30 days after discharge between non-ARDS group and real ARDS group

The age and incidence of hypertension were significantly higher in the real ARDS group (56.4 ± 13.0 years and 31 (93.9%), respectively) than in the non-ARDS group (52.0 ± 11.58 years and 132 (73.7%), respectively) (P < 0.05,Table 6). The time from onset to operation was significantly lower in the real ARDS group (14.0 h [9.0-24.5]) than in the non-ARDS group (20.0 h [11.0–76.0]) (P < 0.05, Table 6). The operation time was significantly longer and the perioperative blood loss, perioperative transfusion (RBC), and perioperative transfusion (plasma) were significantly higher in the real ARDS group (420.0 h [349.0-541.5], 3621.0 mL [2393.0-7575.0], 10.0 U [6.0-14.5], 1200.0 mL [800.0-2450.0], respectively) than in the non-ARDS group (357.0 h [310.0-420.0], 2254.0 mL [1847.0-2950.0], 6.0 U [3.0–10.0], 1000.0 mL [740.0-1310.0], respectively) (P < 0.05; Table 6). The lactate, incidence of AKI and cerebral infarction, APACHE II score and mortality at 30 days after discharge were significantly higher and the mechanical ventilation time, ICU stay, and post-operative hospital stay were significantly longer in the real-ARDS group (4.8mmol/L(3.4–8.4), 13.0(39.4%), 6.0(18.2%),18.0 score [14.5–24.0], 4 [12.1%], 117.0 h [82.0-178.0], 8.0 days [6.0–12.0], 18.0 days [15.0–23.0], respectively) than in the non-ARDS group (3.1mmol/L(2.2–4.8), 33.0(18.4%), 8.0(4.5%), 14.0 score [12.0–16.0], 4 cases [2.2%], 41.0 h [28.0-64.3], 4.0 days (3.0–6.0), 14.0 days (11.0–19.0), respectively) (P < 0.05, Table 7). In-hospital mortality slightly increased from 2.2% in the non-ARDS group to 12.1% in the real-ARDS group (P = 0.064; Table 7). The risk of death within 30 days after discharge was higher in the real ARDS group than in the non-ARDS group (log-rank test, P < 0.05; Fig. 4). Cox survival analysis was used to adjust for age, hypertension, and onset to operation time; the risk of death within 30 days after discharge in the real ARDS group still remained significantly higher than that in the non-ARDS group (HR: 4.633; 95% CI: 1.012–21.202; P = 0.048, Table 8).

**Table 1:** Clinical characteristics and perioperative data of normal oxygenation group, simple hypoxemia group, and ARDS group after surgery

Variable	All patients	Normal oxygenation	Simple hypoxemia	ARDS	*P*-value
	(n = 212)	(n = 51, 24.1%)	(n = 98, 46.2%)	(n = 63, 29.7%)	
Age (year, M [IQR])	53.0 (44.3–62.0)	53.0 (41.0–59.0)	51.0 (44.0–62.0)	55.0 (46.0–66.0)	0.238
Male (n, %)	149 (70.3)	36 (70.6)	72 (73.5)	41 (65.1)	0.523
BMI (kg/m^2^,‾x±s)	26.4±4.1	24.5±3.8	27.0±3.9	27.1±4.1	0.001^a^
Smoking (n, %)	78 (36.8)	20 (39.2)	32 (32.7)	26(41.3)	0.498
Drinking (n, %)	59 (27.8)	17 (33.3)	24 (24.5)	18 (28.6)	0.514
Hypertension (n, %)	163 (76.9)	28 (54.9)	74 (75.5)	61 (96.8)	< 0.001^b^
Diabetes (n, %)	10 (4.7)	1 (2.0)	4 (4.1)	5 (7.9)	0.310
CHD (n, %)	31 (14.6)	7 (14.3)	14 (14.3)	10 (15.9)	0.941
Stroke (n, %)	15 (7.1)	5 (9.8)	5 (5.1)	5 (7.9)	0.443
CKD (n, %)	6 (2.8)	1 (2.0)	2 (2.0)	3 (4.8)	0.574
Onset to operation time (h, M [IQR])	19.0 (11.0-53.8)	25.0 (13.0-144.0)	20 (10.0-72.8)	16 (9.0–27.0)	0.027^c^
Hypoxemia (n, %)	76 (35.8)	6 (11.8)	40 (40.8)	30 (47.6)	< 0.001^d^
Shock (n, %)	9 (4.2)	1 (2.0)	3 (3.1)	5 (7.9)	0.275
WBC (10^9^/L, M [IQR])	11.7 (8.7–15.1)	9.5 (7.8–13.1)	12.3 (9.10–15.1)	11.8 (8.8–15.8)	0.009^e^
NLR (M [IQR])	0.11 (0.07–0.20)	0.12 (0.07–0.30)	0.10 (0.07–0.19)	0.10 (0.07–0.20)	0.331
PLT (10^9^/L, M [IQR])	176.0 (145.0-215.8)	175.0 (144–218)	182.0 (149.8-215.3)	172.0 (139.0-221.0)	0.720
Hemoglobin (g/L,‾x±s)	132.7 ± 18.5	129.7 ± 17.8	133.5 ± 18.8	134.1 ± 18.6	0.391
PT (s, M [IQR])	12.5 (11.8–13.6)	12.7 (12.2–13.9)	12.4 (11.8–13.5)	12.6 (11.6–13.4)	0.178
Fibrinogen (g/L, M [IQR])	2.2 (1.8-3.0)	2.5 (1.8–3.3)	2.2 (1.8-3.0)	2.2 (1.9–2.8)	0.553
ALT (U/L, M [IQR])	27.0 (20.0-38.8)	24.0 (18.0-34.3)	27.0 (21.75-41)	27.0 (18.1–46.0)	0.106
Bilirubin (µmol/L, M [IQR])	15.7 (12.0-22.3)	15.2 (11.3–22.3)	15.7 (12.7–21.1)	15.7 (10.6–22.8)	0.562
Surgical method Total arch replacement (n, %) Hemiarch replacement (n, %)	201(94.8) 11(5.2)	44(86.3) 7(13.7)	94(95.9) 4(4.1)	63(100) 0(0)	0.002^f^
Creatinine (µmol/L, M [IQR])	73.6 (62.5–90.7)	69.2 (56.3–86.1)	72.6 (62.9–90.7)	78.9 (65.6-100.8)	0.100
Operation time (min, M [IQR])	360.0 (312.8-439.3)	355.0 (300.0-420.0)	354 (309.8-418.5)	385.0 (340.0-530.0)	0.002^ g^
CBP time (min, M [IQR])	180.7 (156.2–221.0)	183.0 (147.0-219.8)	174.2 (156.9-218.8)	184.9 (159.0-240.0)	0.306
ACCT (min, M [IQR])	103.5 (83.1-127.4)	106.0 (91–140)	105.2 (81.7-127.1)	100.0 (80.0-124.0)	0.313
Central body temperatu(℃,M[IQR])	28.9 (28.3–29.6)	29.3 (28.4–29.7)	28.9 (28.2–29.6)	2 8.8 (28.4–29.5)	0.208
Perioperative blood loss (mL, M [IQR])	2448.0 (1877.8–3441.0)	2065.0 (1711.0-2850.0)	2252.5 (1845.8-3081.8)	2878.0 (2166.0-5303.0)	< 0.001^ h^
Perioperative transfusion (RBC, U, M [IQR])	6.0 (4.0–10.0)	6.0 (2.0–8.0)	6.0 (3.8–8.5)	8.0 (6.0–14.0)	0.002^i^
Perioperative transfusion (plasma, mL, M [IQR])	1000.0 (800.0-1400.0)	800.0 (600.0-1200.0)	1000.0 (800–1305.0)	1150.0 (800.0-1800.0)	< 0.001^j^
Perioperative fluid load(ml, M [IQR])	-849.5(-1709.5-511.3)	-712.0(-1535.0-427.0)	-1010.0(-1933.0-272.8)	-1400.0(-1829.0-1287.0)	0.168

PT: prothrombin time ; BMI: body mass index; IQR: interquartile range; CHD: coronary heart disease; CKD: chronic kidney disease; WBC: white blood cell; NLR: neutrophil–lymphocyte ratio; PLT: platelet count; CPB: cardiopulmonary bypass; ACCT: aortic cross clamp time; RBC: red blood cell.

a: ARDS vs. normal oxygenation (P = 0.001), normal oxygenation vs. simple hypoxemia (P < 0.001); b: ARDS vs. normal oxygenation (P < 0.001), ARDS vs. simple hypoxemia (P < 0.001), normal oxygenation vs. simple hypoxemia (P = 0.01);

c: ARDS vs. normal oxygenation (P = 0.022);

d: ARDS vs. normal oxygenation (P < 0.001), normal oxygenation vs. simple hypoxemia (P < 0.001);

e: ARDS vs. normal oxygenation (P = 0.023), normal oxygenation vs. simple hypoxemia (P = 0.014);

f: ARDS vs. normal oxygenation (P = 0.008);

g: ARDS vs. normal oxygenation (P = 0.014), ARDS vs. simple hypoxemia (P = 0.004);

h: ARDS vs. normal oxygenation (P < 0.001), ARDS vs. simple hypoxemia (P = 0.001);

i: ARDS vs. normal oxygenation (P = 0.006), ARDS vs. simple hypoxemia (P = 0.006);

j: ARDS vs. normal oxygenation (P = 0.001).

**Table 2:** Multivariable regressions analysis for the effects of pre-operative hypoxemia on the risk of simple hypoxemia and ARDS after surgery

Variable	Model I (OR, 95 CI %)		Model II (OR, 95 CI %)		Model III (OR, 95CI %)	
	Simple hypoxemia ARDS		Simple hypoxemia ARDS		Simple hypoxemia ARDS	
Pre-operative hypoxemia	5.17 (2.02–13.27)	6.28 (2.55–18.27)	4.00 (1.53–10.58)	4.70 (1.67–13.25)	4.81 (1.67–13.81)	8.51 (2.64–27.47)
*P*-value	0.01	< 0.01	0.005	0.003	0.004	< 0.01

Model I, unadjusted variable; Model II, adjusted for BMI and hypertension; Model III, adjusted for BMI, hypertension, onset to operation time, pre-operative leukocyte, operation time, surgical method and perioperative blood loss.

**Table 3:** Clinical outcomes of normal oxygenation group, simple hypoxemia group, and ARDS group after surgery

Variable	All patients	Normal oxygenation	Simple hypoxemia	ARDS	*P-*value	
	(n = 212)	(n = 51)	(n = 98)	(n = 63)		
Lactate(mmol/L,M [IQR])	3.4(2.4–5.1)	2.8(2.0-3.6)	3.3(2.3–4.9)	4.6(3.1-7.0)	< 0.001^a^	
OI(M [IQR])	132.0 (100.0-300.0)	304.0 (301.0-309.0)	130.0 (110.0-161.3)	96.0 (88.0-114.0)	< 0.001^b^	
AKI (n, %)	46(21.7)	4(7.8)	19(19.4)	23(36.5)	0.001^c^	
Liver insufficiency (n, %)	60(28.3)	13(25.5)	25(25.5)	22(34.9)	0.380	
Cerebral infarction(n, %)	14(21.7)	2(3.9)	3(3.1)	9(4.3)	0.013^d^	
re-tracheal intubation	3(1.4)	0(0)	0(0)	3(4.8)	0.039	
APACHE II (score, M [IQR])	14.0 (12.0–17.0)	12.0 (11.0–14.0)	14.0 (12.0–16.0)	17.0 (14.0–21.0)	< 0.001^e^	
Mechanical ventilation time (h, M [IQR])	43.0 (31.0-80.5)	36.5 (23.8.0–45.0)	40.0 (30.8–57.1)	86.0 (57.3–158.0)	< 0.001^f^	
ICU stay length (day, M [IQR])	5.0 (3.0–7.0)	4 0.0(3.0–6.0)	4.0 (3.0-5.3)	7.0 (6.0-11.5)	< 0.001^ g^	
Post-operative hospital stay length (day, M [IQR])	15.0 (12.0–20.0)	15.0 (11.0–18.0)	13.0 (11.0-17.3)	18.0 (14.0-24.5)	< 0.001^ h^	
In-hospital mortality (n, %)	3 (1.4)	1 (0.2)	0 (0)	2 (3.2)	0.158	
Mortality rate at 30 days after discharge (n, %)	8 (3.7)	2 (3.9)	2 (2.0)	4 (6.3)	0.371	

a: ARDS vs. normal oxygenation (P < 0.001), ARDS vs. simple hypoxemia (P = 0.001);

b: ARDS vs. normal oxygenation (P < 0.001), ARDS vs. simple hypoxemia (P < 0.001), normal oxygenation vs. simple hypoxemia (P < 0.006);

c: ARDS vs. normal oxygenation (P < 0.001);ARDS vs. simple hypoxemia(p = 0.016)

d: ARDS vs. simple hypoxemia(p = 0.019)

e: ARDS vs. normal oxygenation (P < 0.001), ARDS vs. simple hypoxemia (P < 0.001), normal oxygenation vs. simple hypoxemia (P = 0.006);

f: ARDS vs. normal oxygenation (P < 0.001), ARDS vs. simple hypoxemia (P < 0.001);

g: ARDS vs. normal oxygenation (P < 0.001), ARDS vs. simple hypoxemia (P < 0.001);

h: ARDS vs. normal oxygenation (P = 0.004), ARDS vs. simple hypoxemia (P < 0.001)

**Table 4:** Clinical characteristics and perioperative data of ARDS patients with pre-operative normal oxygenation and pre-operative hypoxemia

Variable	ARDS group		*P*-value
	Preoperative normal oxygenation n = 33 (52.4%)	Preoperative hypoxemia n = 30 (47.6%)	
Age (year, ‾x±s)	56.4 ± 13.0	53.3 ± 11.8	0.330
Male (n,%)	21 (63.6)	20 (66.7)	0.801
BMI (kg/m^2^,M [IQR])	26.0 (23.9–28.5)	27.1 (24.8–30.4)	0.271
Smoking (n, %)	13 (39.4)	13 (43.3)	0.751
Drinking (n, %)	10 (30.3)	8 (26.7)	0.750
Hypertension (n, %)	31 (93.9)	30 (100.0)	0.493
Diabetes (n, %)	3 (9.1)	2 (6.7)	1.00
CHD (n, %)	6 (18.2)	4 (13.3)	0.857
Stroke (n, %)	3 (9.1)	2 (6.7)	1.00
CKD (n, %)	0 (0.0)	3 (10.0)	0.204
Onset to operation time (h, M [IQR])	14.0 (9.0-24.5)	18.0 (11.8–38.5)	0.148
Shock (n, %)	2 (6.1)	3 (10.0)	0.912
WBC (10^9^/L, ‾x±s)	11.9 ± 3.8	13.9 ± 5.4	0.101
NLR (M [IQR])	0.12 (0.07–0.23)	0.10 (0.07–0.19)	0.901
PLT (10^9^/L, ‾x±s)	174.7 ± 56.7	183.6 ± 59.6	0.545
Hemoglobin (g/L,‾x±s)	132.2 ± 16.8	136.2 ± 20.5	0.397
PT (s, M [IQR])	12.8 (11.9–13.7)	12.1 (11.3–13.3)	0.173
Fibrinogen (g/L, M [IQR])	2.2 (1.9–2.6)	2.5 (1.8-3.0)	0.328
ALT (U/L, M [IQR])	27.0 (16.2–42.0)	29.5 (18.8–55.7)	0.389
Bilirubin(µmol/L,M [IQR])	15.7 (11.5–23.3)	16.4 (9.7–22.5)	0.929
Creatinine(µmol/L,M(IQR))	76.5 (62.8–90.0)	85.4 (66.1-118.3)	0.318
Operation time (min, M (IQR))	420.0 (349.0-541.5)	367.5 (338.3-490.5)	0.113
CBP time (min, M [IQR])	184.9 (162.9–251.0)	185.0 (156.1-235.3)	0.836
ACCT (min, M [IQR])	105.0 (81.5-124.9)	93.5 (76.4-114.8)	0.250
Central body temperatu(℃,M[IQR])	28.6(28.3–29.5)	29.1(28.6–29.6)	0.162
Perioperative blood loss (mL, M [IQR])	3621.0 (2393.0-7575.0)	2492.0 (2010.8–3732.0)	0.010
Perioperative transfusion (RBC, U, M [IQR])	10.0 (6.0-14.5)	7.0 (4.0-10.3)	0.113
Perioperative transfusion (plasma, mL, M [IQR])	1200.0(800.0-2450.0)	1030.0 (882.5-1607.5)	0.345
Perioperative fluid load(ml, ‾x±s)	210.0(-1637.0-1533.0)	-233.5(-1829.0-418.0)	0.425

**Table 5:** Clinical outcomes of ARDS patients with pre-operative normal oxygenation and pre-operative hypoxemia

Variable	ARDS group		*P*-value
	Pre-operative normal oxygenation n = 33 (52.4%)	Preoperative hypoxemia, n = 30 (47.6%)	
Lactate(mmol/L,M [IQR])	4.8(3.4–8.4)	3.8(2.8–5.6)	0.025
OI(M [IQR])	100.0(87.5–121.0)	96.0(89.5-106.5)	0.444
AKI (n, %)	13.0(39.4)	10.0(33.3)	0.618
Liver insufficiency (n, %)	12.0(36.4)	10.0(33.3)	0.801
Cerebral infarction(n, %)	6.0(18.2)	3.0(10.0)	0.571
re-tracheal intubation	2.0(6.1)	1.0(3.3)	1.000
APACHE II (score, M [IQR])	18.0 (14.5–24.0)	16.0 (14.0–18.0)	0.049
Mechanical ventilation time (h, M [IQR])	117.0 (82.0-178.0)	70.5 (42.0-122.3)	0.023
ICU stay length (day, M [IQR])	8.0 (6.0–12.0)	7.0 (5.0-11.25)	0.252
Pos-toperative hospital stay length (day, M [IQR])	18.0 (15.0–23.0)	19.0 (12.0–30.0)	0.942
In-hospital mortality (n, %)	2 (6.1)	0 (0.0)	0.493
Mortality rate at 30 days after discharge (n, %)	4 (12.1%)	0 (0%)	0.146

**Table 6:** Clinical characteristics and perioperative data of non-ARDS and real ARDS groups

Variable	Non-ARDS, n = 179 (84.4%)	Real ARDS, n = 33 (15.6%)	*P*-value
Age (year, ‾x±s)	52.0 ± 11.58	56.4 ± 13.0	0.047
Male(n,%)	128 (71.5)	21 (63.6)	0.363
BMI (kg/m^2^,‾M [IQR])	26.8 (23.9–29.1)	25.9 (23.9–28.5)	0.914
Smoking (n, %)	65 (36.3)	13 (39.4)	0.736
Drinking (n, %)	49 (27.4)	10 (30.0)	0.864
Hypertension (n, %)	132 (73.7)	31 (93.9)	0.011
Diabetes (n, %)	7 ( 3.9)	3 (9.1)	0.399
CHD (n, %)	25 (14.0)	6 (18, 2)	0.718
Stroke (n, %)	12 (6.7)	3 (9.1)	0.903
CKD (n, %)	6 (3.4)	0 (0)	0.593
Onset to operation time(h, M [IQR])	20 (11.0–76.0)	14.0 (9.0-24.5)	0.016
Shock (n, %)	7 (3.9)	2 (6.1)	0.926
WBC (10^9^/L, M [IQR])	11.7 (8.7–15.1)	11.8 (8.7–15.2)	0.941
NLR (M [IQR])	0.11 (0.07–0.20)	0.12 (0.07–0.23)	0.787
PLT (10^9^/L, M [IQR])	177.0 (145.0-218.0)	172.0 (139.0-209.0)	0.537
Hemoglobin (g/L,‾x±s)	132.8 ± 18.8	132.2 ± 16.8	0.844
PT (s, M [IQR])	12.5 (11.8–13.6)	12.8 (11.9–13.7)	0.561
Fibrinogen (g/L, M [IQR])	2.2 (1.8–3.1)	2.2 (1.9–2.6)	0.245
ALT (U/L, M [IQR])	27.0 (20.6–38.0)	27.0 (16.2–42.0)	0.577
Bilirubin (µmol/L, M [IQR])	15.7 (12.0-21.9)	15.7 (11.5–23.3)	0.956
Creatinine (µmol/L, M [IQR])	72.6 (62.5–91.1)	76.5 (62.8–90.0)	0.546
Surgical method			
Total arch replacement (n, %)	168.0(93.9)	33.0(100.0)	0.300
Hemiarch replacement (n, %)	11.0(6.1)	0(0)	
Operation time (min, M [IQR])	357.0 (310.0-420.0)	420.0 (349.0-541.5)	0.001
CBP time (min, M [IQR])	180.0 (155.1-220.7)	184.9 (162.9–251.0)	0.248
ACCT (min, M [IQR])	103.0 (83.3–128.0)	105.0 (81.5-124.9)	0.952
Perioperative blood loss (mL, M [IQR])	2254.0 (1847.0-2950.0)	3621.0 (2393.0-7575.0)	< 0.001
Perioperative transfusion (RBC, U, M [IQR])	6.0 (3.0–10.0)	10.0 (6.0-14.5)	0.001
Perioperative transfusion (plasma, mL, M [IQR])	1000.0 (740.0-1310.0)	1200.0(800.0-2450.0)	0.013
Perioperative fluid load(ml, M [IQR])	-900(-1712.0-392.0)	210(-1800.5-1659.0)	0.096

**Table 7:** Clinical outcomes of non-ARDS group and real ARDS group

Variable	Non-ARDS n = 179 (84.4%)	Real ARDS n = 33 (15.6%)	*P*-value
Lactate(mmol/L,M [IQR])	3.1(2.2–4.8)	4.8(3.4–8.4)	< 0.001
OI(M [IQR])	140.0(106.0-301.0)	100.0(87.5–121.0)	< 0.001
AKI (n, %)	33.0(18.4)	13.0(39.4)	0.007
Liver insufficiency (n, %)	48.0(26.8)	12.0(36.4)	0.263
Cerebral infarction(n, %)	8.0(4.5)	6.0(18.2)	0.004
re-tracheal intubation	1.0(0.6)	2.0(6.1)	0.064
APACHE II (score, M [IQR])	14.0 (12.0–16.0)	18.0 (14.5–24.0)	< 0.001
Mechanical ventilation time (h, M [IQR])	41.0 (28.0-64.3)	117.0 (82.0-178.0)	< 0.001
ICU stay length (day, M [IQR])	4.0 (3.0–6.0)	8.0 (6.0–12.0)	< 0.001
Post-operative hospital stay length (day, M [IQR])	14.0 (11.0–19.0)	18.0 (15.0–23.0)	0.001
In-hospital mortality(n, %)	1 (0.6)	2 (6.1)	0.064
Mortality rate at 30 days after discharge (n, %)	4 (2.2)	4 (12.1)	0.025

**Table 8:** Cox proportional hazard ratio model for 30 days after discharge

Variable	HR	95% CI	*P-*value
Real ARDS	4.633	1.012–21.202	0.048
Age (per year)	1.036	0.972–1.104	0.274
Hypertension	0.743	0.086–6.444	0.787
Onset to operation time (per minute)	1.000	0.992–1.009	0.914

## Discussion

Under-recognition of ARDS is now attributed in part to the significant clinical variability reported among individuals who match standard ARDS criteria. Identifying ARDS subphenotypes is one way to untangle the clinical complexities. It’s critical to uncover relevant but currently unrecognized subgroups of ARDS that is embraced by the wide mainstream definition [13]. This study has two advantages over previous studies. (1) After surgery, hypoxic patients with Stanford type A aortic dissection were firstly separated into two groups: simple hypoxia and ARDS. The link between pre-operative and post-operative hypoxia was investigated. (2)After surgery, ARDS with Stanford type A aortic dissection was firstly separated into two groups: ARDS with pre-operative normal oxygenation and ARDS with pre-operative hypoxemia.

This study revealed that pre-operative and post-operative hypoxemia was a common complication in patients with AAD.post-operative hypoxemia included simple hypoxemia and ARDS. Univariate logistic regression analysis results suggested pre-operative hypoxemia as a risk factor for post-operative simple hypoxemia and ARDS. After adjusting potential confounders, pre-operative hypoxemia was still a risk factor for post-operative simple hypoxemia and ARDS. There are no studies describing the risk factors of post-operative hypoxemia comprising simple hypoxemia and ARDS. Previous studies [6, 19, 25, 26] suggested that pre-operative hypoxemia was a risk factor for post-operative hypoxemia or ARDS. Post-operative hypoxemia should include patients with simple hypoxemia and those with ARDS. This study is helpful to clarify the exact relationship between pre-operative hypoxemia and post-operative ARDS.

The APACHE II score was significantly higher and the mechanical ventilation time and ICU and post-operative hospital stays were significantly longer for the ARDS group than those for patients with simple hypoxia and normal oxygenation. There was no significant difference in the above indices between patients with simple hypoxia and normal oxygenation after surgery. These results indicated that the disease severity was significantly higher in ARDS patients than in patients with simple hypoxia and normal oxygenation. Patients with simple hypoxia did not show increased disease severity as compared with patients with normal oxygenation. However, there was no significant increase in in-hospital mortality or the risk of death within 30 days of discharge among patients with ARDS (log-rank test). Su et al. [20]. also found that post-operative ARDS after AAD was associated with the risk of post-operative complications, but did not increase the risk of in-hospital death or 3-year death. Although this study and previous studies [20, 26] had shown that ARDS did not increase the risk of death, patients with ARDS are critically ill and have longer mechanical ventilation time and ICU stay that increase the risk of ventilator-related lung injury and nosocomial infection as well as the cost of hospitalization. Therefore, early intervention to eliminate the risk factors associated with post-operative ARDS and reduce the incidence of post-operative ARDS has important clinical significance. This study and another previous study [19] found that ARDS did not increase the risk of death, thus contradicting our previous knowledge about ARDS.

In this study, post-operative ARDS patients were divided into two groups, pre-operative hypoxemia and pre-operative normal oxygenation. The results showed that perioperative blood loss and APACHE II score were significantly higher, and the mechanical ventilation time was significantly longer in the ARDS group with pre-operative normal oxygenation than in the ARDS group with pre-operative hypoxemia. Thus, ARDS patients with pre-operative normal oxygenation had more severe disease. Moreover, the risk of death within 30 days of discharge was marginally higher for ARDS patients with pre-operative normal oxygenation than ARDS patients with pre-operative hypoxemia (log-rank test, P = 0.051). There has been no relevant study until now. Therefore, we considered that post-operative ARDS patients with pre-operative normal oxygenation may not be real ARDS patients. Hence, all patients were divided into real ARDS group (ARDS with pre-operative normal oxygenation) and non-ARDS group (ARDS with pre-operative hypoxemia, post-operative simple hypoxemia, and post-operative normal oxygenation). Interestingly, onset to operation time is statistically significant inverse relation between real ARDS group and non-ARDS group. This might be related to that the shorter the time from onset to operation, the lower the degree of thrombus, which leads to intraoperative hemostasis difficulty and the longer time. The real ARDS group had significantly higher lactate value, incidence of AKI, APACHE II score and longer mechanical ventilation time and ICU and post-operative hospital stays than the non-ARDS group. In-hospital mortality was marginally higher (P = 0.064) and the risk of death within 30 days of discharge was significantly higher for real ARDS patients (log-rank test). Cox survival analysis after adjusting for potential confounding factors showed that the risk of death within 30 days after discharge among real ARDS patients still remained significantly higher than that among non-ARDS patients. It must be pointed out that pre-operative hypoxemia did not exclude the occurrence of real ARDS after surgery. This study data shown that most post-operative ARDS patients with pre-operative hypoxemia were imitators of real ARDS, and the real ARDS patients were few. This study emphasized that ARDS patients with pre-operative normal oxygenation were severe, and they deserved more attention from clinicians. ARDS patients with pre-operative hypoxemia cannot simply be identified as low-risk patients. That will be a very meaningful topic how we diagnose or screen real ARDS patients in this subset(post-operative ARDS with pre-operative hypoxemia). A new, more rigorous diagnostic criterion may be needed to diagnose these patients. At present, the mechanism of pre-operative hypoxemia with AAD patients remains unclear, and the in-depth research on the mechanism of AAD hypoxemia is conducive to the solution of this topic.

This study was a retrospective single-center case-control study. Considering the very low 30d mortality (6.3%) in the study, the second misclassification probability should decline, that is the sample size should be enlarged. However, AAD is an infrequent disease. Though we retrospected the data of the center for 6 years, only 212 patients were included. The increase of the sample size would prolong the retrospective time and cause recall bias. The study subjects will be inconsistent. The results may be affected by non-randomization. The pathogenesis and operation of AAD are complex, and there may be some unknown variables potentially affecting the development of ARDS that we could not fully include in the study. The residual confounding and bias could be inevitable.

## Conclusion

This study confirmed that pre-operative hypoxemia was an independent risk factor for post-operative simple hypoxemia and ARDS, and that there was no significant increase in the nosocomial mortality and the risk of death within 30 days after discharge among ARDS patients. Evaluation of the clinical outcomes of ARDS patients with pre-operative normal oxygenation and ARDS patients with pre-operative hypoxemia surprised us that ARDS patients with pre-operative hypoxemia were less severe. They may belong to different subgroups. Therefore, in this study, patients were further divided into real ARDS (postoperative ARDS with pre-operative normal oxygenation) and non-ARDS (postoperative ARDS with pre-operative hypoxemia and post-operative the simple hypoxemia and post-operative normal oxygenation) groups, which confirmed that the real ARDS group was more severe and had significantly higher risk of death within 30 days after discharge than the non-ARDS group. The in-hospital mortality of real ARDS patients was marginally higher than that of non-ARDS patients, a difference that nearly reached statistical significance.

## Electronic supplementary material

Below is the link to the electronic supplementary material.

Supplementary Material 1: Table 1. The Berlin Definition of Acute Respiratory Distress Syndrome [14].

## Data Availability

All relevant datasets are available from the corresponding author upon reasonable request.

## Abbreviations

AAD: Stanford type A aortic dissection
ACCT: aortic cross clamp time
ALT: Alanine aminotransferase
ANOVA: analysis of variance
ARDS: Acute respiratory distress syndrome
BMI: Body mass index
CHD: Coronary heart disease
CI: Confdence interval
CKD: Chronic kidney disease
CPB: Cardiopulmonary bypass
FiO2: Fraction of inspired oxygen
HR: Hazard ratio
IQR: Interquartile range
NLR: Neutrophil–lymphocyte ratio
OI: Oxygenation index
OR: Odds ratios
PaO2: Partial pressure of arterial oxygen
PLT: Platelet count
PT: Prothrombin time
RBC: Red blood cell
ROC: Receiver operating characteristics
SD: Standard deviation
SPO2: Peripheral capillary oxygen saturation
WBC: White blood cell
