# Supplementary material for: Effect of pre-operative hypoxemia on the occurrence and outcomes of post-operative ARDS in Stanford type a aortic dissection patients
Source: Respir Res. 2023 Jun 17;24:161. doi: 10.1186/s12931-023-02457-8 (PMC10276407; doi:10.1186/s12931-023-02457-8)
Supplement: Supplementary file 1 — Supplementary Material 1: Table 1. The Berlin Definition of Acute Respiratory Distress Syndrome [14]. [file 12931_2023_2457_MOESM1_ESM.doc]

Supplementary table 1

The Berlin Definition of Acute Respiratory Distress Syndrome[14]

| Acute Respiratory Distress Syndrome |
| --- |
| Timing Within 1 week of a known clinical insult or new or worsening  respiratory symptoms |
| Chest imaging^a^ Bilateral opacities—not fully explained by effusions, lobar/lung  collapse, or nodules |
| Origin of edema Respiratory failure not fully explained by cardiac failure or fluid overload. Need objective assessment (eg,echocardiography) to exclude hydrostaticedema if no risk factor present |
| Oxygenation^b^ PaO2/FIO2＜300 mm Hg with PEEP or CPAP≥5 cm H2O^c^ |

Abbreviations: CPAP, continuous positive airway pressure; FIO2, fraction of inspired oxygen; PaO2, partial pressure of arterial oxygen; PEEP, positive end-expiratory pressure.

a: Chest radiograph or computed tomography scan.

b: If altitude is higher than 1000 m, the correction factor should be calculated as follows: [PaO2/FIO2×(barometric pressure/ 760)].

c：This may be delivered noninvasively in the mild acute respiratory distress syndrome group.

[14]ARDS Definition Task Force et al. “Acute respiratory distress syndrome: the Berlin Definition.” JAMA vol. 307,23 (2012): 2526-33
